# A dynamically interacting flexible loop assists oligomerisation of the *Caenorhabditis elegans* centriolar protein SAS-6

**DOI:** 10.1038/s41598-019-40294-2

**Published:** 2019-03-05

**Authors:** Julia M. C. Busch, Michèle C. Erat, Iris D. Blank, Maria Musgaard, Philip C. Biggin, Ioannis Vakonakis

**Affiliations:** 1 University of Oxford, Department of Biochemistry, Oxford, OX1 3QU United Kingdom; 2 Present Address: University of Warwick, Mathematical Institute, Coventry, CV4 7AL United Kingdom; 3 Present Address: University of Ottawa, Department of Chemistry and Biomolecular Sciences, Ottawa, ON K1N 6N5 Canada

## Abstract

Centrioles are conserved organelles fundamental for the organisation of microtubules in animal cells. Oligomerisation of the spindle assembly abnormal protein 6 (SAS-6) is an essential step in the centriole assembly process and may act as trigger for the formation of these organelles. SAS-6 oligomerisation is driven by two independent interfaces, comprising an extended coiled coil and a dimeric N-terminal globular domain. However, how SAS-6 oligomerisation is controlled remains unclear. Here, we show that in the *Caenorhabditis elegans* SAS-6, a segment of the N-terminal globular domain, unresolved in crystallographic structures, comprises a flexible loop that assists SAS-6 oligomerisation. Atomistic molecular dynamics simulations and nuclear magnetic resonance experiments suggest that transient interactions of this loop across the N-terminal dimerisation interface stabilise the SAS-6 oligomer. We discuss the possibilities presented by such flexible SAS-6 segments for the control of centriole formation.

## Introduction

Centrioles are conserved organelles widespread in the eukaryotic kingdom^1–3^. In animals, a pair of centrioles comprise the structured core of centrosomes, which direct formation of the microtubule network and the mitotic spindle during cell division^4,5^. In this capacity, centrioles are crucial for controlling the overall cell architecture, facilitating intracellular cargo transport, anchoring the endoplasmic reticulum and the Golgi apparatus, and ensuring the equitable segregation of genetic material during mitosis. Furthermore, centrioles in all eukaryotic lineages except fungi and higher plants also act close to the membrane, where, as basal bodies, they template formation of microtubule-based cilia and flagella^6^. In this manner, centrioles are essential for diverse aspects of cellular behaviour including locomotion via flagellar and cillial beating, and sensing, via the antena-like primary cillium. Unsurprisingly, given the wide swath of cellular processes dependant on centrioles, mutations in genes coding for essential components of these organelles are linked to major human genetic disorders and diseases, including male sterility, ectopic pregnancies, multisystemic ciliopathies, primary microcephaly and potentially cancer^7–11^.

The formation of new centrioles is a highly regulated process which occurs once per cycle in dividing cells^12–15^. The main molecular features of the centriole assembly pathway are conserved^13,14^, and involve the initial localisation at the site of assembly of the coiled coil protein SPD-2 in *Caenorhabditis elegans* via interactions with the protein SAS-7^16^, followed by the kinase ZYG-1 and SAS-6. Structural and functional studies of SAS-6 have revealed that this protein assists in establishing the canonical radial symmetry of centrioles^17^, thereby influencing a key element of the overall organelle architecture. SAS-6 forms large 9-fold symmetric oligomers *in vitro*^18–21^ that bear striking resemblance to scaffold-like assemblies observed at the centre of centrioles, the ‘cartwheels’, which are believed to seed formation of these organelles^17,22^. Disruption of SAS-6 oligomerisation directly abrogates the canonical pathway of centriole formation^18,20,23^, while SAS-6 variants engineered to form oligomers with symmetry other than 9-fold were seen to influence the organelle radial symmetry^24^. Thus, a broad consensus has emerged placing SAS-6 oligomerisation as a crucial molecular event at the onset of centriole assembly.

The mechanisms by which SAS-6 oligomerisation is controlled in cells remain, however, poorly understood. At the molecular level, oligomerisation is driven by two independent dimerisation interfaces on SAS-6, comprising a long, parallel, dimeric coiled-coil (the CC interface) and a dimeric globular domain at the protein N-terminus (the NN interface)^18–20,23,25,26^. Interactions across both of these interfaces are essential for SAS-6 oligomer formation; however, whereas the CC interface is relatively stable (K_d_ ~1 μM)^18^ and readily forms SAS-6 dimers in the cell cytoplasm^27^, the N-terminal dimer is significantly weaker (K_d_ ~50–100 μM in most systems)^18,20^, thereby presenting a challenge for the assembly of stable SAS-6 oligomers in cells^28^. SAS-6 is co-recruited to the site of centriole assembly and interacts with the protein SAS-5 in *C. elegans*^25,26,29^, while in insects and vertebrates binding to SAS-6 is similarly reported for the proteins Ana2^30,31^ and STIL^32,33^, respectively. SAS-5, Ana2 and STIL self-associate into hexameric (SAS-5) ^34,35^ or tetrameric (Ana2/STIL)^23,36^ complexes, and these complexes have been suggested to assist SAS-6 oligomerisation in cells via an avidity mechanism, whereby multiple weak interactions act cooperatively^23,34,35,37,38^.

SAS-6 binding to Ana2/STIL depends on phosphorylation of these proteins by the Plk4 kinase, the vertebrate and insect analogue of ZYG-1. This dependence offers a putative mechanism for control of SAS-6 oligomerisation in insects and vertebrates via modulation of the Ana2/STIL–SAS-6 interaction affinity, and hence ‘fine tuning’ of the aforementioned avidity effect. In *C. elegans*, however, a model system for centriole cell biology, no such direct modulation of the SAS-5–SAS-6 interaction has been observed, although SAS-5 protein levels and targeting to the site of centriole assembly are reportedly controlled by the PP2A phosphatase^39–41^. Instead, earlier studies suggested that direct SAS-6 phosphorylation by the ZYG-1 kinase at a specific amino acid, S123, triggers centriole formation and ensures that SAS-6 is stably incorporated in the organelle^42^. Interestingly, S123 locates at the SAS-6 N-terminal domain, and its phosphorylation was proposed to affect the NN interface dimerisation affinity and, thus, the propensity of SAS-6 to oligomerise^26^. However, a later study convincingly demonstrated using S123 substitutions that phosphorylation of this SAS-6 residue is not required for *C. elegans* centriole formation^43^.

Nevertheless, the molecular logic of modulating the SAS-6 NN dimerisation affinity in order to control oligomer formation remains a strong one. Compared to the SAS-6 coiled-coil dimer, which spans hundreds of amino acids^18^, the N-terminal dimer principally depends on the interaction of a single amino acid, I154 in *C. elegans*, with a hydrophobic cavity across the NN dimerisation interface^18,20^. In this manner, it offers an attractive target for a relatively small, trigger-like molecular event to exert maximum influence on the oligomerisation propensity of SAS-6. Furthermore, we noted that a substantial segment of the *C. elegans* SAS-6 N-terminal domain, which includes S123, remained unresolved in all crystallographic structures of this domain to date. Thus, we set out to explore the effect of this *C. elegans* SAS-6 segment on the protein properties.

Here, we report that *C. elegans* SAS-6 features a ~30-amino acid flexible loop that does not have a counterpart in the algal, insect or vertebrate SAS-6 variants studied to date. The location and length of this loop allow it to transiently interact with multiple amino acids across the NN dimerisation interface, and these transient but frequent interactions cumulatively stabilise formation of SAS-6 oligomers. We note that many SAS-6 variants, including those from several species of human-infective parasites, feature similar, presumed flexible, insertions, and we discuss their possible role as elements controlling the trigger of centriole assembly.

## Results

### *C. elegans* SAS-6 features a long, flexible loop in its N-terminal domain

The *C. elegans* SAS-6 N-terminal domain (henceforth, *Ce*SAS-6_N_) has been the subject of previous X-ray crystallographic studies that resolved the structures of the wild-type (WT) protein^18^ as well as derivatives^25^. In both cases an extended segment of this domain connecting α-helix 2 (α2) and β-strand 5 (β5), spanning amino acids 105–128 of *Ce*SAS-6_N_, was absent from the structures as no electron density could be observed for the corresponding residues. The α2-β5-connecting segment was, thus, presumed disordered and, in the interest of efficient protein crystallisation, was removed from subsequent *Ce*SAS-6 crystallographic efforts by excising residues I103 to P130 from the protein expression constructs. The resulting Δ103–130 variant of *Ce*SAS-6_N_ remained folded and showed only minimal structural changes compared to the WT protein as judged by X-ray crystallography (Cα RMSD of 0.3 Å)^25^; nuclear magnetic resonance (NMR) ^15^N heteronuclear single quantum coherence (HSQC) spectra of *Ce*SAS-6_N_ variants also showed a very high degree of similarity, suggesting limited long-range structural changes to this domain as a result of modifications (Supplemental Fig. 1).

**Figure 1:**
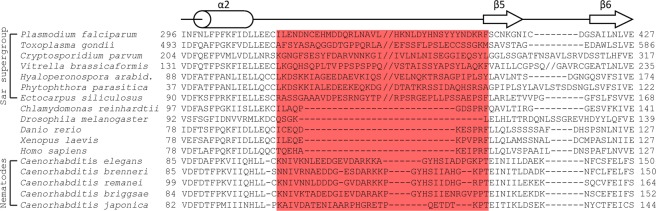
A α2-β5 insertion is common in nematode and pathogen SAS-6 proteins. Shown here is a sequence alignment focused on the α2-β6 region of SAS-6 proteins. The secondary structure of *Ce*SAS-6 is represented schematically at the top. The α2-β5 segment shown to be flexible in *C. elegans* (K101-T131) is highlighted in red. Double slash (‘//’) marks denote areas were 5 or more amino acids have been removed for clarity. Sequences were aligned manually using crystallographic (*C. elegans, C. reinhardtii, D. melanogaster, D. rerio*)^18,20,23^ or predicted SAS-6 structures as guides. Structure predictions were performed by Phyre2^68^. Sequences derive from UniProt accession numbers C6KSS4 (*P. falciparum*), A0A0F7V199 (*T. gondii*), Q5CPW9 (*C. parvum*), A0A0G4ERZ9 (*V. brassicaformis*), M4B318 (*H. arabidopsis*), W2PGE7 (*P. parasitica*), D7FSC1 (*E. siliculosus*), A9CQL4 (*C. reinhardtii*), Q9VAC8 (*D. melanogaster*), Q7ZVT3 (*D. rerio*), Q6NRG6 (*X. laevis*), Q6UVJ0 (*H. sapiens*), O62479 (*C. elegans*), G0N6C0 (*C. brenneri*), E3NH99 (*C. remanei*), Q60P76 (*C. briggsae*) and A0A2H2I8W7 (*C. japonica*).

A similarly extended amino acid segment between α2 and β5 was not observed in the structures of *Chlamydomonas reinhardtii* (green algae)^18^, fruit fly^23^ or zebrafish^20^ SAS-6 N-terminal domains, and sequence alignments suggest it is similarly absent from the human and frog variants (Fig. 1). However, we noted that SAS-6 proteins from the Sar eukaryotic supergroup^44^, which includes several animal and plant pathogen species such as the malaria parasite *Plasmodium falciparum*, feature an extended segment connecting α2 and β5 that, in the case of *P. falciparum*, spans approximately 90 amino acids. Thus, this feature of SAS-6 is not restricted to nematode sequences but is likely also present in other branches of the eukaryotic kingdom.

**Figure 2:**
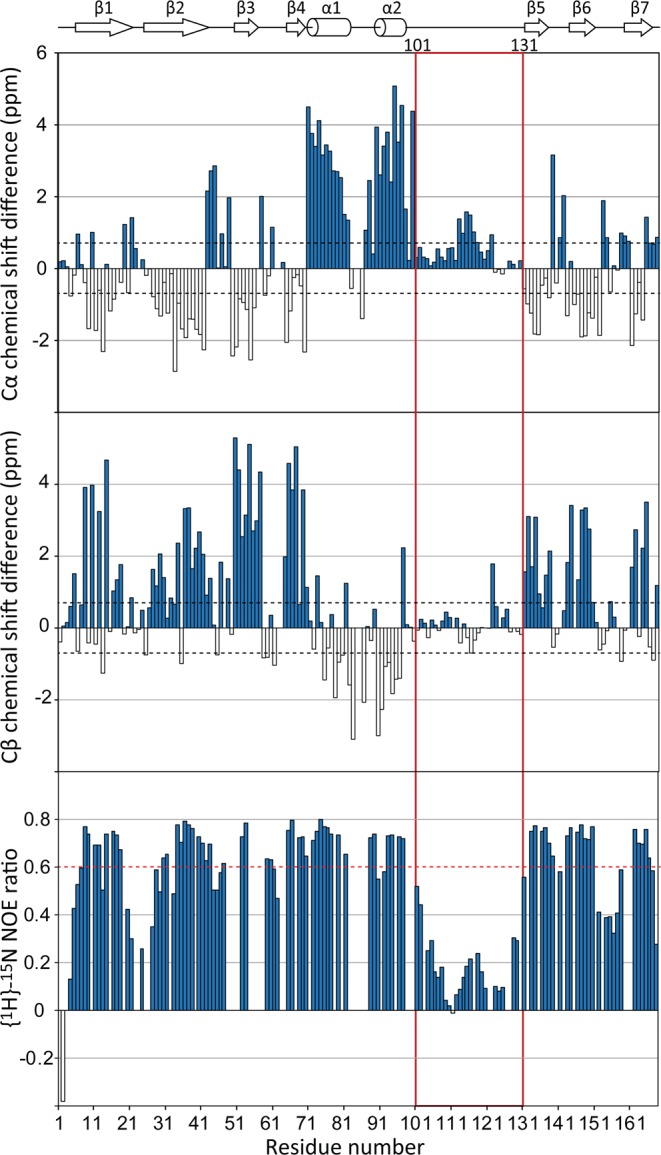
The *Ce*SAS-6 α2-β5 loop is flexible and unstructured. Shown here are per amino acid NMR chemical shift differences from random coil of *Ce*SAS-6_N_ Cα (top panel) and Cβ (middle panel) atoms, as well as {^1^H}-^15^N NOE ratios from the same amino acids (bottom panel). The *Ce*SAS-6_N_ secondary structure elements inferred from the crystallographic structure of this domain^18^ are represented schematically at the top. Black dashed lines (top and middle panels) denote thresholds over which the chemical shift differences are considered to support the presence of stable secondary structure elements^45^. A red dashed line (bottom panel) denotes a threshold below which {^1^H}-^15^N NOE ratios suggest that amino acids have substantial high-frequency (sub-ns timescale) motions^46^.

To characterise the structural state of the *Ce*SAS-6 α2-β5-connecting segment we employed NMR, which can provide residue-specific information on amino acid properties in solution. In particular, the NMR chemical shifts of amino acid Cα and Cβ atoms are sensitive to the protein secondary structure, and display characteristic patterns of deviation from random coil chemical shift values depending on the type of secondary structure present in a given protein sequence^45^. We observed that the Cα and Cβ chemical shifts of *Ce*SAS-6_N_ amino acids showed patterns consistent with the secondary structure elements revealed by X-ray crystallography of this domain (Fig. 2A,B); however, chemical shift deviations from random coil were small at the α2-β5 segment, suggesting that this region of the protein lacks stable secondary structure elements.

Furthermore, we analysed the *Ce*SAS-6_N_ amino acid mobility using heteronuclear {^1^H}-^15^N NOE NMR experiments, which are sensitive to motions in the picosecond to nanosecond time scale. Values of {^1^H}-^15^N NOE ratios over 0.6 are considered as indicative of structured protein segments, whereas NOE ratio values lower than that correspond to protein regions of increasing mobility^46^. As seen in Fig. 2C, {^1^H}-^15^N NOE ratios in *Ce*SAS-6_N_ support the rigid state of secondary structure elements observed by crystallography, whereas loops connecting secondary structure elements, such as between β1–β2, β2–β3 and β6–β7, are more mobile. Strikingly, the *Ce*SAS-6_N_ α2-β5 segment shows evidence of very high mobility, with {^1^H}-^15^N NOE ratios lower than 0.6 for a continuous span of amino acids between K101 and T131. We conclude that *Ce*SAS-6_N_ features a ~30 amino acid-long, flexible loop connecting α2 with β5, removal of which does not compromise the folded state of *Ce*SAS-6_N_.

### The α2-β5 loop is necessary for CeSAS-6 oligomerisation

**Figure 3:**
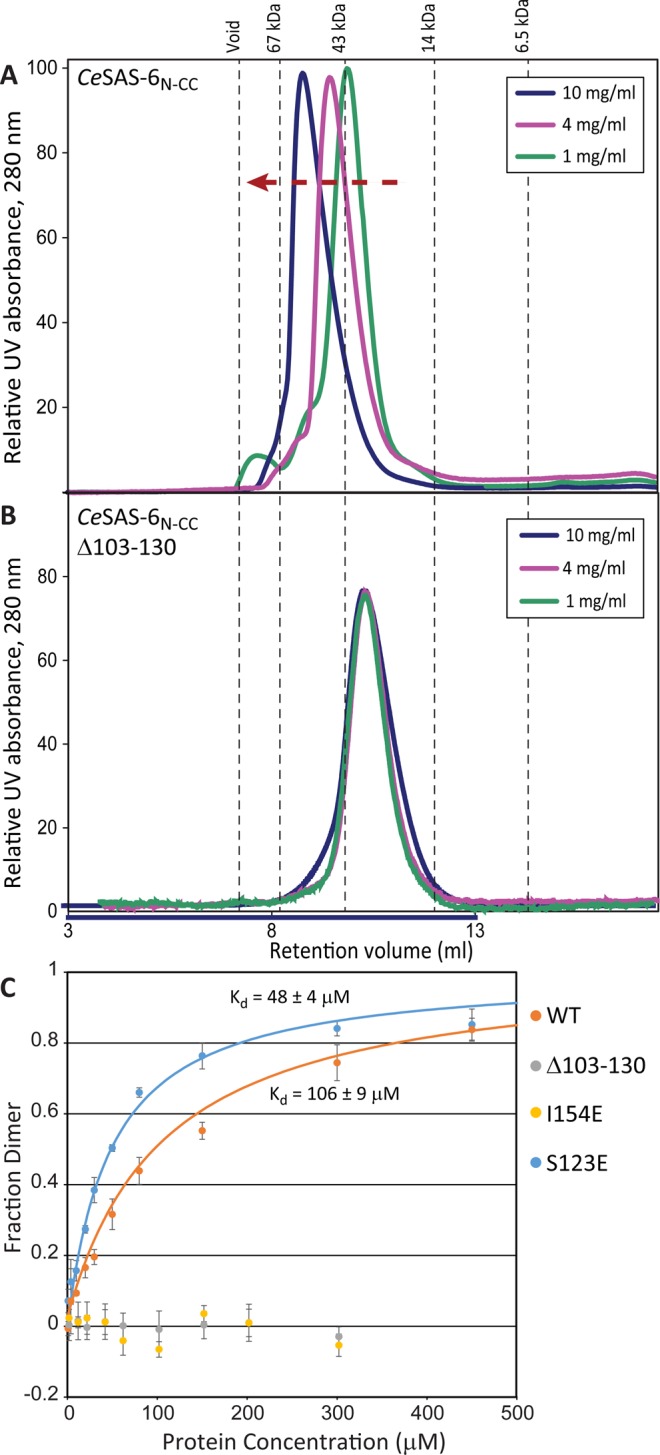
The *Ce*SAS-6 α2-β5 loop supports NN protein dimerisation. (**A**,**B**) Size-exclusion chromatography traces showing the elution profiles of *Ce*SAS-6_N-CC_ WT (**A**) or Δ103–130 (**B**) variants at different protein concentrations. WT *Ce*SAS-6_N-CC_ elutes at smaller retention volumes as protein concentration increases, indicating formation of larger oligomers. In contrast, *Ce*SAS-6_N-CC_ Δ103–130 does not form large oligomers under the same conditions. The apparent molecular masses of standard samples passed through the same size-exclusion column are shown as dashed lines. For reference, the calculated molecular weight of the *Ce*SAS-6_N-CC_ WT dimer is 48.8 kDa. (**C**) Fluorescence polarisation-monitored titrations of 1,5-IAEDANS-conjugated *Ce*SAS-6_N_ WT and variants as function of protein concentration. Points and error bars represent means and standard deviations, respectively, derived from three independent experiments. Solid lines denote fits of ideal self-association models to the data, with the estimated dissociation constants (K_d_) shown. Raw fluorescence polarisation data were converted to fractions of *Ce*SAS-6 NN dimers formed using the maximum polarisation change estimated from the fits.

SAS-6 oligomerisation is a defining property of this protein that is essential for canonical centriole assembly^18,20,23^; thus, we assessed the impact of the α2-β5 loop in the ability of *Ce*SAS-6 to form oligomers. We performed analytical size exclusion chromatography (SEC) experiments using a *Ce*SAS-6 construct that included both the N-terminal domain as well as a short stretch of the coiled coil (*Ce*SAS-6_N-CC_). In previous studies WT and variants of *Ce*SAS-6_N-CC_ were observed to form stable dimers mediated by the CC interface, which then assembled into large oligomers in a concentration-dependent manner via the NN interaction^25^. Consistent with these previous results, SEC experiments showed increased apparent molecular size of *Ce*SAS-6_N-CC_ WT as function of protein concentration as judged by the reduction in elution volume from the SEC column (Fig. 3A). In contrast, a similar protein construct lacking the α2-β5 loop (*Ce*SAS-6_N-CC_ Δ103–130) showed no increase in apparent molecular size beyond the CC interface-mediated CeSAS-6_N-CC_ dimer, even in concentrations as high as 10 mg/ml (Fig. 3B). We surmised that the α2-β5 loop contributes to the formation of large *Ce*SAS-6 oligomers by strengthening the NN dimerisation of this protein.

To confirm this observation, we examined the dimerisation propensity of the *Ce*SAS-6 N-terminal domain in isolation. Quantitative interaction assays using *Ce*SAS-6_N_ WT site-specifically labelled with 1,5-IAEDANS showed increased fluorescence polarisation as function of protein concentration, consistent with the formation of *Ce*SAS-6_N_ dimers mediated by the NN interface with a K_d_ of approximately 100 μM (Fig. 3C). In contrast, a *Ce*SAS-6_N_ I154E variant, which lacks the hydrophobic residue critical for NN dimerisation^18^, showed no increase in fluorescence polarisation in these assays. Similarly, *Ce*SAS-6_N_ Δ103–130 did not produce changes in fluorescence polarisation under the same conditions, suggesting that *Ce*SAS-6 NN dimerisation affinity is greatly weakened in the absence of the α2-β5 loop. Thus, both SEC and fluorescence polarisation experiments independently support the role of loop α2-β5 in strengthening NN dimerisation and, hence *Ce*SAS-6 oligomerisation.

### Simulations and NMR reveal transient interactions formed by the α2-β5 loop

**Figure 4:**
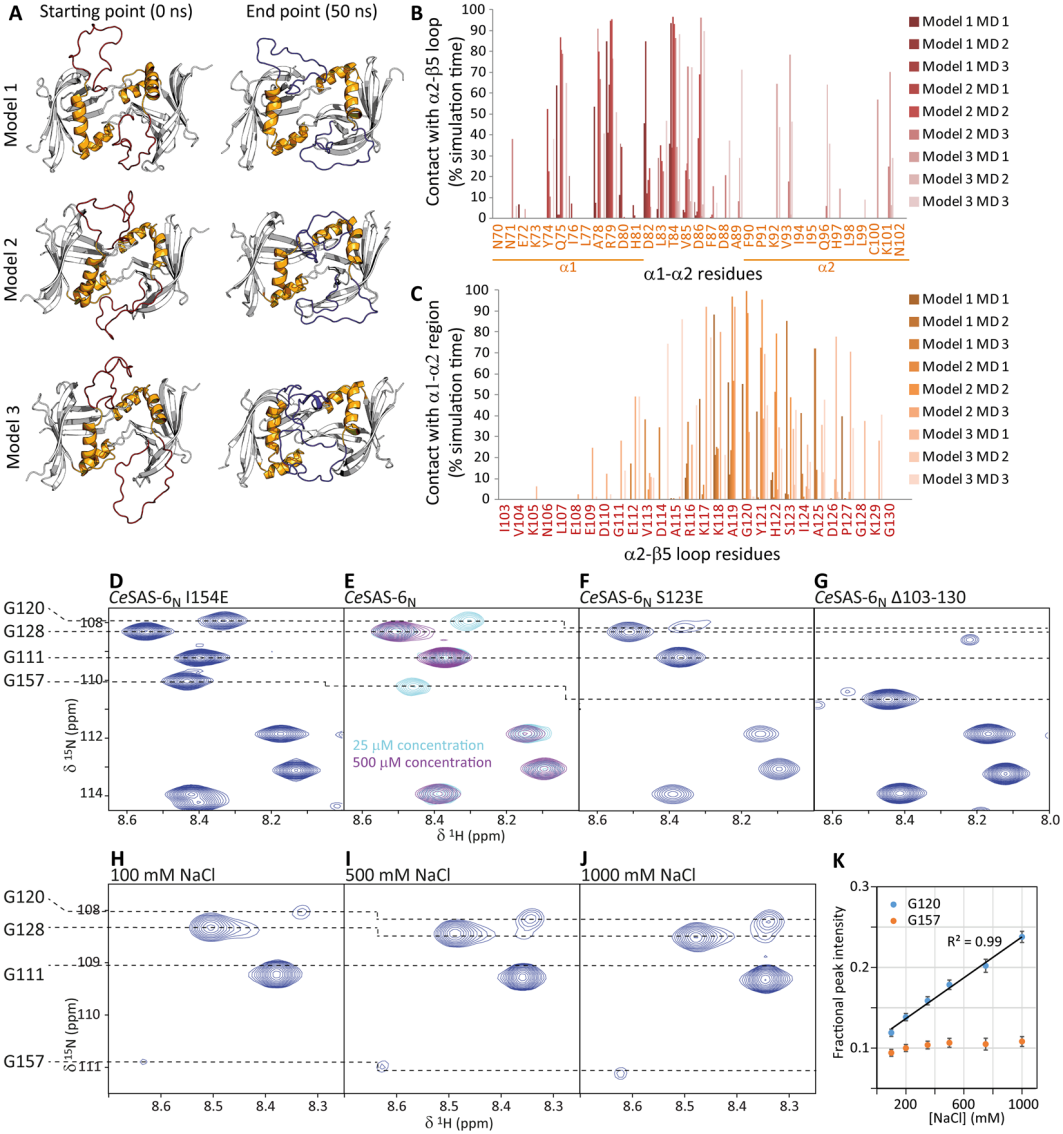
The *Ce*SAS-6 α2-β5 loop forms transient contacts across the NN dimerisation interface. (**A**) Snapshots of three structural models of *Ce*SAS-6_N_ that include the α2-β5 loop in different starting conformations, at the beginning (0 ns) and end (50 ns) of representative atomistic MD simulations. The α2-β5 loop is coloured red at the starting and blue at the end point of simulations; the α1-α2 helices are shown in gold. Note that in all cases the α2-β5 loop forms extensive contacts with the structured core of *Ce*SAS-6_N_ during the MD simulations. (**B**,**C**) Quantitative analysis of contacts between α1-α2 residues and the α2-β5 loop (**B**), and *vice versa* (**C**), in nine MD simulations (three simulations per starting *Ce*SAS-6_N_ dimer model). Contacts are expressed as fraction of simulation time during which residues are in close proximity (distance <3.5 Å) to α1-α2 helices (**C**) or the α2-β5 loop (**B**). The amino acid sequences are shown, as is the position (**B**) of α1-α2 helices within the sequence. (**D**–**J**) Sections of NMR ^1^H-^15^N HSQC spectra of (**D**) the monomeric *Ce*SAS-6_N_ I154E variant at 500 μM protein concentration, (**E**) *Ce*SAS-6_N_ WT at 25 (light blue) or 500 μM (purple) protein concentration, (**F**) *Ce*SAS-6_N_ S123E at 500 μM protein concentration and (**G**) the *Ce*SAS-6_N_ Δ103–130 variant at 500 μM protein concentration. (**H**–**J**) NMR spectra of *Ce*SAS-6_N_ at 500 μM concentration and different amounts of NaCl as shown. The resonances of G111, G120, G128 and G157 amino acids are indicated. Note that all four glycine resonances are strong in the monomeric *Ce*SAS-6_N_ I154E variant (**D**) as is also the case for G157 in the *Ce*SAS-6_N_ Δ103–130 variant (**F**). In contrast, in *Ce*SAS-6_N_ WT or S123E the G120 and G157 resonances disappear as function of protein concentration, indicating the formation of μs-ms time scale contacts by these residues. At high ionic strength conditions the G120 resonance increases in intensity, suggesting that loop α2-β5 forms fewer contacts. (**K**) Fractional intensities of the G120 and G157 resonances as function of NaCl concentration. Intensities were normalised to those of the G111 and G128 resonances in the same spectra. Error bars derive from the spectral signal-to-noise ratios. The G120 resonance intensity is fit to a linear regression model with the indicated R^2^.

We proceeded to analyse how the α2-β5 loop strengthens *Ce*SAS-6 NN dimerisation using atomistic molecular dynamics (MD) simulations. The NN-mediated dimer is well defined in the *Ce*SAS-6_N_ crystallographic structures; however, these structures did not resolve the α2-β5 loop and, thus, cannot provide starting positions for the loop amino acids for computational simulations. For that reason, we constructed models of *Ce*SAS-6_N_ dimers where the α2-β5 loop residues were placed in energetically favourable but variable arrangements. We derived three different models of *Ce*SAS-6_N_ dimers with distinct α2-β5 loop conformations for each monomeric subunit, and performed nine, 50 ns-long MD simulations (three simulations starting from each *Ce*SAS-6_N_ dimer model) to explore the available structural landscape. The starting and end points of one MD simulation for each *Ce*SAS-6_N_ dimer are shown in Fig. 4A. We observed that in all cases the α2-β5 loop of *Ce*SAS-6_N_ rearranges to form interactions with helices α1-α2 of the opposing *Ce*SAS-6_N_ monomer. Notably, these interactions were not stable; rather the α2-β5 loops continuously repositioned over α1-α2 during the course of simulations, breaking and reforming interactions with several residues therein. The α2-β5 loop conformations did not stabilise even when simulations were extended to 100 ns length, suggesting that loop mobility observed in simulations reflects the flexibility of this protein segment shown by NMR experiments.

To quantitatively compare the α2-β5 loop conformations across different simulations we evaluated the length of time during which loop amino acids are in close proximity (<3.5 Å distance) to residues of the α1-α2 region as proportion of the total MD simulation time. As shown in Fig. 4B, in most simulations one or more α2-β5 loop amino acids contact the C-terminus of helix α1 and the α1-α2 linker for over 50% of MD time; residues of helix α2 are also contacted in a minority of cases. Similar analysis showed that the α2-β5 loop region primarily involved in α1-α2 contacts spans amino acids R116-A125, which locate approximately at the middle of the α2-β5 loop (Fig. 4C). We note that the majority of contact residues in both the α1-α2 region and the α2-β5 loop are hydrophilic in nature, including a large number of charged amino acids; indeed, these residues primarily form hydrogen bond and electrostatic interactions in the MD simulations.

Analysis of ^1^H-^15^N HSQC NMR spectra supports the formation of transient contacts by residues at the middle of the α2-β5 loop. Specifically, we used the glycine amino acids of the α2-β5 loop, which are easily distinguished in NMR spectra (Fig. 4D–G), as probes to quickly ascertain the structural state of loop residues. In the monomeric *Ce*SAS-6_N_ I154E protein variant (Fig. 4D) these glycine residues (G111, G120 and G128) always give rise to strong resonances, as is also the case for WT *Ce*SAS-6_N_ at low concentrations (25 μM) when the protein is mostly monomeric (Fig. 4E, light blue). In contrast, at high (500 μM) protein concentrations, when WT *Ce*SAS-6_N_ forms NN-mediated dimers, the NMR resonance of G120 nearly disappears (Fig. 4E, purple), indicating that this residue at the middle of the α2-β5 loop is involved in μs-ms timescale interactions. A similar effect is seen for the resonance of G157 at high *Ce*SAS-6_N_ concentrations, as this amino acid is located directly at the *Ce*SAS-6 NN dimerisation interface. In contrast, we observed no perturbation of the G111 or G128 resonances regardless of protein concentration. To assess whether the transient contacts of the α2-β5 loop are electrostatic in nature we titrated NaCl to samples of WT *Ce*SAS-6_N_ at high protein concentration (500 μM). Under these conditions, the G120 increases in intensity as function of ionic strength (Fig. 4H–K), suggesting that the α2-β5 loop forms fewer μs-ms timescale interactions. This is consistent with electrostatic contacts of the α2-β5 loop being masked by increased NaCl amounts. In contrast, the G157 resonance intensity is virtually unchanged upon NaCl titration, suggesting that *Ce*SAS-6_N_ remains dimeric. We conclude that formation of the *Ce*SAS-6 NN dimer causes residues at the middle of the α2-β5 loop, including G120, to engage in intermediate timescale electrostatic interactions, as suggested by the MD simulations.

### Interactions of the α2-β5 loop stabilise the *Ce*SAS-6 NN dimer

**Figure 5:**
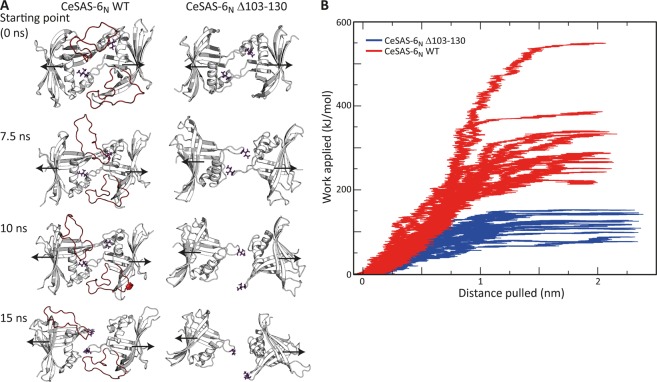
The α2-β5 loop stabilises *Ce*SAS-6_N_ dimerisation in steered MD simulations. (**A**) Snapshots of *Ce*SAS-6_N_ WT and Δ103–130 dimers at different time points during representative steered MD simulations. The α2-β5 loop is coloured red and amino acid I154, which directly mediates *Ce*SAS-6 NN dimerisation, is shown as sticks. The forces applied on the *Ce*SAS-6_N_ monomeric subunits are represented by black arrows as reference. (**B**) Graphic representation of work applied to separate the *Ce*SAS-6_N_ monomeric subunits versus distance pulled in steered MD simulations. Shown here are 14 independent simulations performed on *Ce*SAS-6_N_ WT (red graphs) and the same number of simulations for *Ce*SAS-6_N_ Δ103–130 (blue graphs).

We proceeded to examine using steered MD simulations whether the transient, interchangeable interactions formed between the α2-β5 loop and the structured core of *Ce*SAS-6_N_ may cumulatively stabilise formation of the *Ce*SAS-6 NN dimer. Pulling forces in opposing directions were applied *in silico* on the monomeric subunits of the *Ce*SAS-6 NN dimer, and the work required to pull the dimer apart was measured during the course of 14 independent simulations for each of *Ce*SAS-6_N_ WT and Δ103–130 variants. We observed that the work necessary for disruption of *Ce*SAS-6 NN dimers varied substantially across different simulations, reflecting the non-equilibrium nature of these experiments; however, in all cases *Ce*SAS-6_N_ Δ103–130 dimers were pulled apart faster and with greater ease compared to *Ce*SAS-6_N_ WT dimers (Fig. 5). A representative example of this is shown in Fig. 5A, where a dimer of *Ce*SAS-6_N_ Δ103–130 has lost all amino acid contacts across the NN interface after 15 ns of simulation time, whereas a dimer of *Ce*SAS-6_N_ WT maintains contacts at the same time point partly through the α2-β5 loop. We surmise that contacts between the α2-β5 loop and the structured core of *Ce*SAS-6_N_ can indeed stabilise the NN dimerisation interface in MD simulations.

To validate the effect of interactions formed by the α2-β5 loop on *Ce*SAS-6 NN dimerisation, we sought to examine whether changes within the loop modulate the NN dimerisation affinity. We chose to modify S123, which locates at the middle section of this loop and forms transient interactions in MD simulations (Fig. 4C). Although S123 phosphorylation has been shown not to have an effect on *C. elegans* centriole assembly^43^, we reasoned that changes at this site might provide an informative *in vitro* tool. Thus, we analysed the effect of a S123E substitution on *Ce*SAS-6_N_ dimerisation using quantitative fluorescence polarisation experiments. Although this substitution does not induce local structural rearrangements or stabilisation of the α2-β5 loop, as judged by heteronuclear {^1^H}-^15^N NOE NMR experiments (Supplemental Fig. 3), we observed that S123E increases NN affinity by approximately 2-fold (Fig. 3C). It is likely that this small but notable change in *Ce*SAS-6 NN affinity upon substituting S123 may have contributed to the lack of clarity on the functional role of this residue in the literature. However, for the purpose of our analysis, this substitution provides evidence that changes in the α2-β5 loop can indeed modulate *Ce*SAS-6 properties.

## Discussion

SAS-6 oligomerisation is a key property for the function of this protein, not least as it assists the establishment of centriolar 9-fold radial symmetry^17,24^. The weakest molecular ‘link’ in SAS-6 oligomerisation is dimerisation of this protein’s N-terminal domain via the NN interface^18–20,23,25^. With this in mind, we examined the properties of a previously unresolved sequence element in the N-terminal domain of *C. elegans* SAS-6. We found that this element, which spans approximately 30 amino acids and connects α2 and β5 of the *Ce*SAS-6 N-terminal domain, is unstructured and highly dynamic in solution (Fig. 2). The flexible nature of this α2-β5 loop is consistent with the lack of electron density for this region in crystallographic structures of the N-terminal domain. Nevertheless, despite the apparent lack of structure, we noted that the α2-β5 loop has a stabilising role in *Ce*SAS-6 NN dimerisation, to the extent that removing this loop abrogated formation of *Ce*SAS-6 oligomers *in vitro* (Fig. 3). The α2-β5 loop enhances *Ce*SAS-6 N-terminal domain dimerisation by forming transient interactions, evident by both NMR and computation (Fig. 4), with the structured core of this domain. Crucially, substituting a single amino acid in the α2-β5 loop further enhanced *Ce*SAS-6 NN dimerisation by approximately 2-fold (Fig. 3C).

The concept of disordered protein segments engaging in, and being important for, protein interactions is widely accepted^47^, and such disordered segments are believed to confer enhanced interaction specificity as well as plasticity. In most cases, disordered segments fold into stable structures upon binding their physiological partner. However, in a subset of protein interactions disordered segments remain unfolded, which has given rise to the notion of ‘fuzzy complexes’ during the last decade^48,49^. Such complexes comprise conformational ensembles even in their functional state, with the relative populations of discrete states within these ensembles subject to change according to the cellular context in order to fine-tune activity. Our work strongly suggests that the *Ce*SAS-6 N-terminal domain forms a fuzzy complex, at least in part, as the α2-β5 loop remains disordered even at the physiologically relevant dimeric state of this domain.

A long α2-β5 loop with high levels of sequence conservation is found in many species of nematode worms, such as throughout the *Caenorhabditis* genus (Fig. 1), despite these species diverging over 30 million years ago^50^. Although our analysis shows that this loop serves to stabilise the CeSAS-6 NN dimer, it is clear from the vertebrate, insect and algal SAS-6 variants lacking this loop that such stabilisation could be achieved more simply by a handful of amino acid changes, not least by replacing I154 with an aromatic amino acid^24,25^. If correct, this raises the question of what is the true purpose of the long α2-β5 loop so that it is maintained across millions of years. We can only speculate the answer to this question; however, a likely clue is offered by the observation that amino acid changes within the α2-β5 loop directly affect the *Ce*SAS-6 NN dimerisation affinity (Fig. 3C). Although phosphorylation of the specific amino acid substituted in our study, S123, is not physiologically relevant^43^, our work demonstrated the principle, common to fuzzy complexes, that even small changes in the disordered segment can affect complex formation. Thus, we propose that the physiological role of the SAS-6 α2-β5 loop in nematode species may be as a modulator of NN dimerisation, and consequently as a molecular control mechanism for regulating SAS-6 oligomerisation. It should be noted that in a multimeric system such as SAS-6 oligomers, where a complex of at least 9 protein dimers is necessary to define centriolar 9-fold symmetry, even small changes in self-association affinity can exert a powerful effect. Indeed, simple simulations suggest that increasing the *Ce*SAS-6 NN dimerisation affinity from 100 μM to 50 μM K_d_ leads to a ~150-fold increase in the likelihood 9 *Ce*SAS-6 dimers associate into an oligomer, and, hence, in the probability that a core structural element of centrioles forms.

Sequence analysis suggests that a large α2-β5 loop is not restricted to nematode SAS-6 proteins, but also found throughout the Sar eukaryotic supergroup (Fig. 1). Of particular interest there are apicomplexan parasites, including *Plasmodium*, *Cryptosporidium* and *Toxoplasma*, that are responsible for widespread and severe human diseases. It will be interesting to examine whether the α2-β5 loop in apicomplexan SAS-6 acts in a similar capacity as in nematode SAS-6 to modulate NN dimerisation. If so, such a behaviour would represent a distinct departure from vertebrate SAS-6 oligomerisation and, thus, may be a mechanism open to exploitation by putative therapeutic agents. Although SAS-6 has only just begun to be studied in these parasites, we note that *Plasmodium* SAS-6 appears essential for malaria transmission^51^.

In conclusion, we report here that a previously uncharacterised loop in the *Ce*SAS-6 N-terminal domain reinforces the self-association interactions of this protein, and that changes in this loop can modulate the formation of large SAS-6 oligomers. As formation of such SAS-6 oligomers is an essential step for the initiation of centriole formation, we postulate that changes in the α2-β5 loop, putatively through yet uncharacterised amino acid modifications, may act as molecular switches that assist in triggering centriole assembly.

## Materials and Methods

### Protein production and purification

*C. elegans* SAS-6 (Uniprot ID 062479) fragments were prepared as described earlier^18,25^; briefly, fragments comprising the protein N-terminal domain (*Ce*SAS-6_N_, amino acids 1–168) or the N-terminal domain plus a short stretch of the coiled-coil interface (*Ce*SAS-6_N-CC_, amino acids 1–215) were cloned in a modified pET15b vector containing an N-terminal His_6_-tag, transformed into *Escherichia coli* BL21 (DE3) cells grown in Luria-Bertani (LB) media, and protein expression was induced for 16 h with 0.25 mM final concentration of isopropylb-D-1-thiogalactopyranoside at 18 °C. Cell pellets were resuspended in lysis buffer containing 20 mM Tris HCl buffer pH 7.5, 500 mM NaCl, 0.5% v/v Triton X-100 and Complete protease inhibitor tablets (Roche), and sonicated for cell lysis. Metal affinity purification of clarified lysates was performed using His-Trap HP columns (GE LifeSciences), followed by His_6_-tag cleavage using thrombin protease (Sigma-Aldrich) and size exclusion chromatography on Sephadex G75 columns (GE LifeSciences) equilibrated in PBS (20 mM sodium phosphate buffer pH 7.0, 150 mM NaCl and 2 mM DTT). For the production of isotopically labelled protein samples *E. coli* cells were grown in M9 minimal media supplemented with ^15^NH_4_Cl and ^13^C_6_-glucose (Isotech) as necessary.

### NMR experiments

Sequence-specific NMR resonance assignments were performed as described previously^52^. Briefly, NMR experiments were performed using Bruker Avance II and Avance III spectrometers with cryogenic TCI probeheads, and 11.7–14.1 T magnetic field strengths. Samples of ^13^C/^15^N-enriched *Ce*SAS-6_N_ S123E I154E variant at 1 mM concentration in PBS buffer were supplemented with 5% v/v D_2_O, 0.02% w/v NaN_3_ and 50 μM 4,4-dimethyl-4-silapentane-1-sulfonic acid. Assignment experiments were performed at 20 °C using 3D CBCA(CO)NH, CBCANH and HNCA pulse sequences. NMR data were processed using NMRpipe^53^ and analysed using PIPP^54^. Assignments were deposited in BioMagResBank under accession number 27607. Chemical shift assignments were transferred to *Ce*SAS-6_N_ WT by overlaying spectra. Spectra overlays were prepared with Sparky^55^. Comparisons of ^13^Cα and ^13^Cβ chemical shifts to those of random coil were performed using the Chemical Shift Index method^56^. Heteronuclear {^1^H}-^15^N NOE experiments were performed in a manner analogous to that described previously^57^.

### Fluorescence polarisation and size exclusion chromatography

Protein samples for fluorescence polarisation were disolved in PBS and featured 1,5-IAEDANS (Invitrogen) fluorescence labels conjugated to C100 of *Ce*SAS-6_N_ using the manusfacturer’s recommended protocol. Measurements were recorded using a PHERASTAR FS fluorimeter (BMG Labtech, λ_ex_ = 340 nm, λ_em_ = 520 nm). Analytical size exclusion chromatography assays were performed using protein samples in PBS and Superdex 75 10/300 GL columns (GE LifeSciences).

### Molecular modelling and all-atom simulations

A complete structure of the *Ce*SAS-6_N_ domain, including residues 103–130 of the α2-β5 loop, was built using Modeller^58^ starting from the crystallographic structure of *Ce*SAS-6_N_ Δ103–130 (RCSB ID 4G79)^25^. 100 models were created, and models for MD simulations were selected visually preferring those structures that minimised clashes in the α2-β5 loop while also lacking secondary structure elements there. Protein models were placed in a 100 × 100 × 100 Å boxes with periodic boundary conditions, and MD simulations were initiated using the all-atom force field AMBER99SB-ILDN^59^ with explicit TIP3P^60^ water molecules and an ionic concentration of 150 mM NaCl. The model was energy minimised using the steepest descent method with a target energy of 100 kJ/(mol nm). For NVT equilibration 200 ps of MD simulations were run with constant temperature at 300 K using a Berendsen thermostat^61^, while applying position restraints for protein heavy atoms. NPT equilibration was achieved by 200 ps of MD simulations in constant pressure of 1 bar using a Berendsen barostat^61^. Position restraints on heavy atoms were removed for production runs of 50 ns, which were started from the same equilibrated starting point but using different seed parameters. All trajectories were generated and analysed with GROMACS v5.02^62^. The distance cut-off for van der Waals and short-range electrostatic interactions was set to 10 Å. Long-range electrostatics were accounted for using the particle mesh Ewald method^63,64^ and the LINCS^65^ algorithm was selected to treat all bonds as constraints, allowing a time step of 2 fs. Residue encounters were calculated using a tcl/tk script and VMD^66^ with a distance cut-off of 3.5 Å.

For steered MD simulations the collective variable (CV) was the distance between the centres of mass of each *Ce*SAS-6_N_ domain monomer. The centres of mass were calculated using the Cα atoms of residues 1–102 and 131–168, thereby excluding residues of the flexible loop. During steered MD simulations the CV was steered towards a distance of 20 Å, which was judged as sufficient to separate the *Ce*SAS-6 NN dimer. Constant velocity of 1 Å/ns and a force constant of 1000 kJ/mol/nm were used. Steered MD simulations were setup and analysed using PLUMED v2.2^67^ and GROMACS v5.02^62^.

## Supplementary information

Supplemental Figures
