# Supplementary material for: A dynamically interacting flexible loop assists oligomerisation of the Caenorhabditis elegans centriolar protein SAS-6
Source: Sci Rep. 2019 Mar 5;9:3526. doi: 10.1038/s41598-019-40294-2 (PMC6401066; doi:10.1038/s41598-019-40294-2)
Supplement: Supplementary file 1 — Supplemental Figures [file 41598_2019_40294_MOESM1_ESM.pdf]

A dynamically interacting flexible loop assists oligomerisation of the *Caenorhabditis elegans* centriolar protein SAS-6

Julia M. C. Busch, Michèle C. Erat, Iris D. Blank, Maria Musgaard, Philip C. Biggin and Ioannis Vakonakis

SUPPLEMENTARY INFORMATION

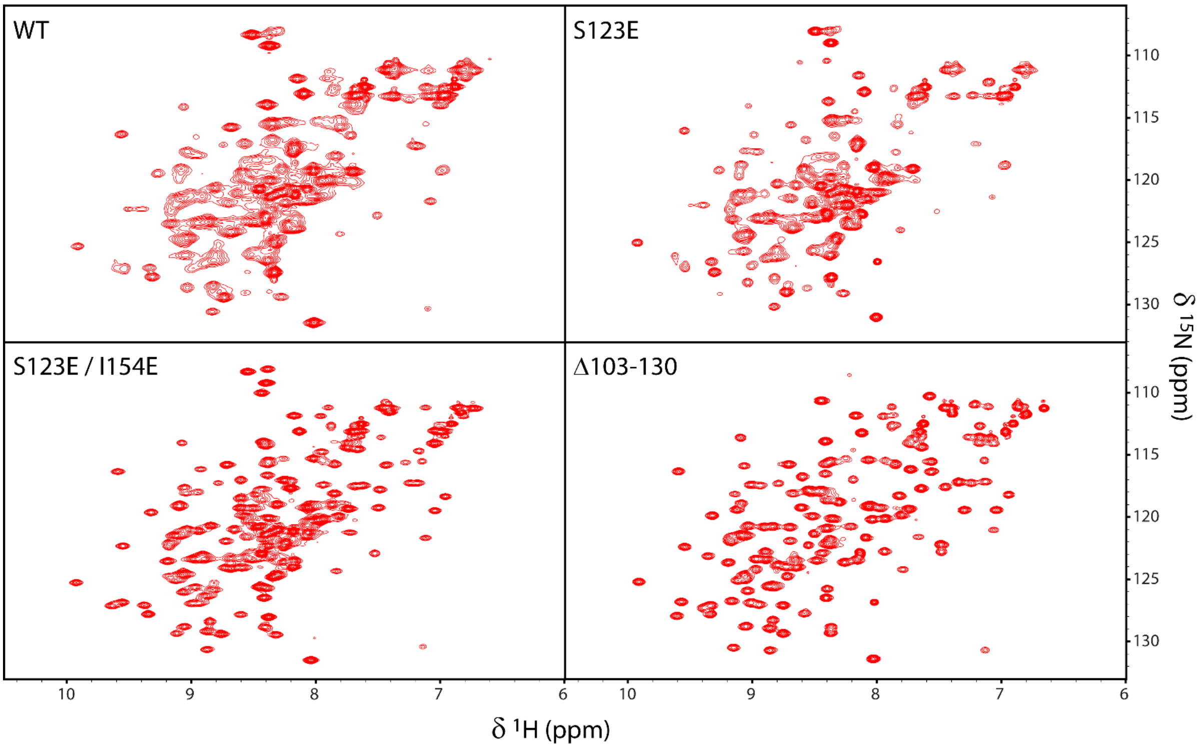

Supplementary Figure 1: The global *Ce*SAS-6$_N$ structure is not perturbed by modifications. Shown here are NMR ^{1}H-^{15}N HSQC spectra of *Ce*SAS-6$_N$ WT and variants as indicated. The overall spectral appearance remains highly similar across these constructs, suggesting that the global structure of this domain is unaffected by modifications. Note that *Ce*SAS-6$_N$ WT and S123E form dimers in solution, which leads to NMR peak broadening due to slower protein tumbling, whereas the S123E / I154E and Δ103-130 variants are monomeric. A number of peaks near the 8.5 ppm ^{1}H resonance frequency visible in the *Ce*SAS-6$_N$ S123E / I154E spectrum are absent in the Δ103-130 spectrum as they correspond to amino acids removed upon deletion of the α2-β5 loop.

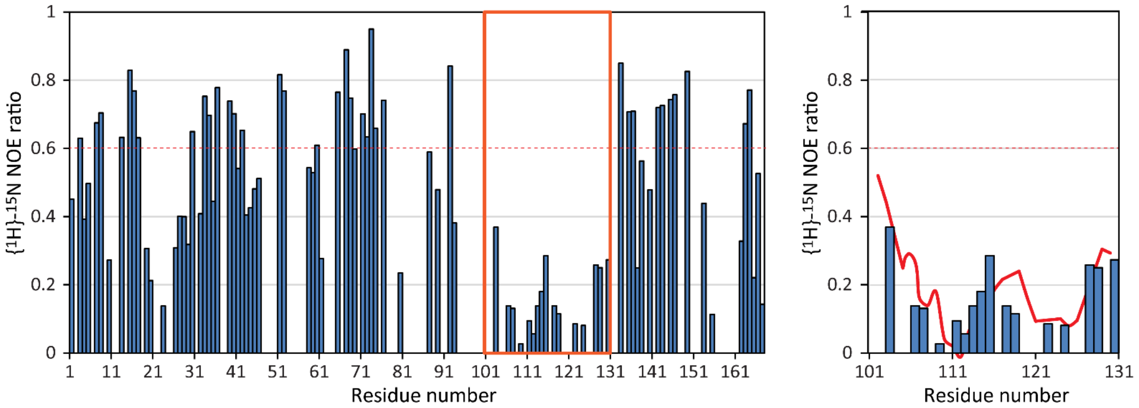

Supplementary Figure 2: The *Ce*SAS-6$_N$ S123E substitution does not stabilise the structure of the α2-β5 loop. Shown here are per amino acid {^{1}H}-^{15}N NOE ratios recorded from the *Ce*SAS-6$_N$ S123E variant. A red dashed line denotes a threshold below which {^{1}H}-^{15}N NOE ratios suggest that amino acids have substantial high-frequency (sub-ns timescale) motions[1]. The boxed area corresponds to amino acids in the α2-β5 loop, and is magnified in the right-hand panel. The same panel shows, as continuous red line, the {^{1}H}-^{15}N NOE ratios recorded from *Ce*SAS-6$_N$ WT (Fig. 2) for the same region of the protein. As shown, the {^{1}H}-^{15}N NOE ratios of WT and S123E variant proteins are highly similar, suggesting that the S123E substitution does not cause a local structuring of the α2-β5 loop.

1) Kay, L. E., Torchia, D. A. & Bax, A. Backbone dynamics of proteins as studied by 15N inverse detected heteronuclear NMR spectroscopy: Application to staphylococcal nuclease. *Biochemistry* **28**, 8972-8979 (1989).
